# Quality evaluation of four hemoglobin screening methods in a blood donor setting along with their comparative cost analysis in an Indian scenario

**DOI:** 10.4103/0973-6247.53874

**Published:** 2009-07

**Authors:** Rashmi Tondon, Anupam Verma, Prashant Pandey, Rajendra Chaudhary

**Affiliations:** *Department of Transfusion Medicine, Sanjay Gandhi Postgraduate Institute of Medical Sciences, Lucknow- 226014, UP, India*

**Keywords:** Blood donation, CuSO4, hemoglobin estimation, HemoCue, HCS, cost analysis

## Abstract

**Background:**

Despite the wide range of methods available for measurement of hemoglobin, no single technique has emerged as the most appropriate and ideal for a blood donation setup.

**Materials and Methods:**

A prospective study utilizing 1014 blood samples was carried out in a blood donation setting for quality evaluation of four methods of hemoglobin estimation along with cost analysis: Hematology cell analyzer (reference), HCS, CuSO4 method and HemoCue.

**Results:**

Mean value of HemoCue (mean ± SD = 14.7 ± 1.49 g/dl) was higher by 0.24 compared to reference (mean ± SD = 13.8 ± 1.52 g/dl) but not statistically significant ( P > 0.05). HemoCue proved to be the best technique (sensitivity 99.4% and specificity 84.4%) whereas HCS was most subjective with 25.2% incorrect estimations. CuSO4 proved to be good with 7.9% false results. Comparative cost analysis of each method was calculated to be 35 INR/test for HemoCue, 0.76 INR /test for HCS and 0.06-0.08 INR /test for CuSO4.

**Conclusion:**

CuSO4 method gives accurate results, if strict quality control is applied. HemoCue is too expensive to be used as a primary screening method in an economically restricted country like India.

## Introduction

Pre-donation hemoglobin screening is among the first and foremost tests done for blood donor selection with the main intention of preventing blood collection from an anemic donor. It is therefore essential, that there should be an accurate and reliable method for hemoglobin determination. According to the Indian Drugs and Cosmetics Act, 1940 for blood donation, the minimum acceptable hemoglobin (Hb) is 12.5 g/dl or hematocrit (Hct) of 38% for both males and females.[1]

There are various methods of hemoglobin estimation which vary from simple paper scale reading to measurement by photometer, each with its own advantages and limitations. The copper sulfate (CuSO_4_) specific gravity method[2] is the traditional method being used for donor screening at many blood centers. Though a cheap and easy method, it does not provide an acceptable degree of accuracy.[3,4] Cyanmethemoglobin method is the method recommended by the International Council for Standardization in Hematology[5] but the main disadvantage is the requirement of venipuncture before the actual donation. The HemoCue test system is a portable, battery-operated photometric device for rapid determination of hemoglobin.[6] The WHO hemoglobin color scale (HCS) is an easy and inexpensive method which measures hemoglobin between 4-14 g/dl in 2 g/dl increments. It is said to provide a reliable indication of presence and severity of anemia where laboratory based hemoglobinometry methods are not available.[7]

Despite the availability of various methods for measuring donor hemoglobin, no single technique has emerged as the most suitable for hemoglobin testing in a blood donation setting. The main objectives of this study were to compare four hemoglobin testing methods and to assess the utility of HCS and HemoCue against a standard hematology analyzer and to ascertain whether any of these methods could replace the traditional copper sulfate method for donor Hb screening. We also sought to ascertain the financial implications of using HemoCue as a primary Hb screening method in an economically restricted country like ours. Though several studies have compared various methods of Hb estimation in blood donors, to our knowledge no studies are available on cost analysis of various methods from resource limited countries.

## Materials and Methods

This prospective study was conducted on 1014 random blood donors attending routine donor sessions at an apex tertiary care hospital based blood center in North India over a period of 6 months (January to June 2005). Two ml of venous blood sample in dipotassium EDTA under identical conditions were drawn from apparently healthy donors after obtaining their consent. Samples were analyzed using four different methods of Hb estimation: Automated hematology cell analyzer (ABX Micros 60, France), HCS, CuSO_4_ specific gravity method and Hemocue (Hemocue B - hemoglobin photometer; Angelholm, Sweden). Testing on CuSO_4_, HCS and HemoCue was done without delay while samples were run on the automated cell analyzer immediately or within 30-60 minutes of collection. To avoid inter-observer variability, blood sampling and analysis of Hb was performed by a single trained operator who first estimated the Hb values by HCS, followed by CuSO_4_, and HemoCue and finally by automated analyzer (reference hemoglobin value). The operator was properly trained on a few pilot samples using the four methods before commencing the study. The working CuSO_4_ solution was prepared (specific gravity 1.053) and standardized every day using standard operating procedure (SOP). The functioning of the HemoCue photometer was checked every day by measuring the control cuvette as per the manufacturer’s instructions. Quality control and calibration of automated hematology analyzer was done as per SOP using manufacturer provided stabilized control reagents. Results were recorded in separate laboratory registers and subsequently transcribed into a SPSS version 12.0 spreadsheet. Results of CuSO_4_ were interpreted as pass or fail at Hb cut-off of ≥ 12.5 g/dl while HCS readings were considered as pass when the readings were ≥ 12.0 g/dl and fail below 12.0 g/dl.

We did the cost analysis by using HemoCue as the primary screening method and compared its cost with the other methods for its implementation in a blood donor setup.

### Statistical Analysis

Statistical analysis was performed using SPSS 12.0 for Windows (Microsoft, Seattle, WA, USA). Sensitivity, specificity, positive predictive value (PPV) and negative predictive value (NPV) of each method was calculated and Bland-Altman plots were drawn for HemoCue and HCS to compare results with automated cell analyzer (gold standard).

## Results

**Figures 1 and 2:**
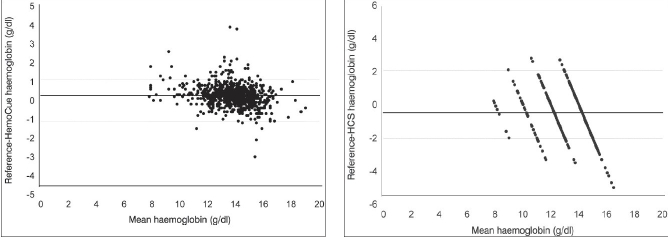
Bland-Altman plots for HemoCue and HCS

The gender distribution of 1014 donor population predominantly consisted of males with only 8% female representation [Table 1]. A total of 167 (16.5%) donors were deferred due to low Hb of which 124 were males (12.2% of the total donors screened). More than half of the prospective female donors were deferred (43/81) because of low Hb levels with 34 (79.07%) having values below 12.0 g/dl. A comparison of different methods used in the present study against the reference hematology analyzer is summarized in Table 2. We assessed the Hb values (mean ± standard deviation) for 1014 venous samples tested with each method. Hb values by HemoCue and HCS showed quite similar results against the reference. However, mean Hb value of HemoCue (14.7 ± 1.49 g/dl) was higher by 0.24 when compared with reference Hb values (13.8 ± 1.52 g/dl). The mean Hb values for HCS were 13.3 ± 1.18 g/dl. HemoCue was found to be most sensitive technique (sensitivity 99.4%; specificity 84.4%). CuSO_4_ also gave good results with overall 7.9% (80/1014) false results with sensitivity of 98.8%, but specificity of 58.1% with a PPV of 92.3% and NPV of 90.7% [Table 2]. The CuSO_4_ screening test inappropriately passed 6.9% (70/1014) donors. Out of these, 65 donors had Hb values between 12.4-12.0 g/dl when tested by reference method [Table 3]. Of the total deferrals, 41 (24.5%) had Hb values below 11.0 g/dl (4.0% of apparently healthy donors). The sensitivity of HCS was 87.2% with 81.8% specificity and 256 samples (25.2%) were incorrectly estimated using this method [Table 4]. On comparing HCS values in 2 g/dl increments, maximum (63.5%) incoherent results were found in the range of 10-12 g/dl. Figures 1 and 2 show graphical representations of Bland-Altman analysis for comparison between two Hb estimation methods i.e. HemoCue and analyzer [Figure 1] and HCS and analyzer [Figure 2] with differences in means and upper and lower 95% limits of agreement (LOA).

**Table 1:** Results of Hb measurement by automated hematology analyzer (ABX Micros 60) reference method (n = 1014)

Gender	Pass	Fail	Total number(%)
Male	829	124	933 (92)
Female	38	43	81 (8)
Total number	847	167	1014
Percentage	83.5	16.5	-

**Table 2:** Comparison of the results obtained by HemoCue, HCS and CuSO4 methods of Hb estimation against the reference (n = 1014)

Result	HemoCue	HCS	CuSO_4_
True positive	842	837	837
True negative	141	45	97
False positive	26	10	70
False negative	05	122	10
Sensitivity (%)	99.4	87.3	98.8
Specificity (%)	84.4	81.8	58.1
Likelihood ratio	630.07	173.69	290.56
Positive predictive value	97.0%	98.8%	92.3%
Negative predictive value	96.6%	26.9%	90.7%
Linear by linear association	794.78	238.59	405.68

**Table 3:** True deferral data (n = 167) from the reference hematology analyzer#

Hb values	True deferral (by analyzer)	Pass by CuSO_4_	Fail by CuSO_4_
7.8-10.9	41	0	41
11.0	4	1	3
11.8	9	1	8
11.9	9	3	6
12.0	20	8	12
12.1	22	11	12
12.2	17	8	11
12.3	29	27	9
12.4	16	11	7
Total	167	70	107

# Results are compared with CuSO_4_ data. Seventy donors were inappropriately accepted while 10 were falsely deferred.

**Table 4:** Comparison of Hb values by HCS against the reference*

Hb values by HCS (g/dl)	Hb values obtained by reference method (g/dl)				
	8 to <10	10 to <12	12 to <14	>14	Total
6 to <8	4	1	0	0	5
8 to <10	4	38	31	0	73
10 to <12	1	27	57	4	89
12 to <14	0	8	511	68	587
>14	0	0	44	216	260
Total	9	74	643	288	1014
643 288 1014 Correctly estimated by HCS	4/9 (44.4%)	27/74 (36.5%)	511/643 (79.5%)	216/288 (75.0%)	
Wrongly estimated by HCS	5/9 (55.6%)	47/74 (63.5%)	132/643 (20.5%)	72/288 (25.0%)	

* Almost 64% of the values were wrongly estimated. The maximum agreement between the two methods seems to be in the higher Hb range (> 14g/dl).

The cost of HemoCue B instrument is about 35000 Indian Rupees (INR) while each disposable microcuvette costs approximately 30 INR. In contrast, copper sulfate powder (500 gm) costs just 175 INR from which 2000-2500 samples can be tested (considering 159.63 gm used for approximately 750-800 tests), thus costing around 0.06-0.08 INR per test. The cost of using HCS was estimated to be 0.76 INR per test (HCS strips for 1000 tests costs 760 INR). Considering an average donor registration of 1700-1800 per month at our centre, the running cost of HemoCue will be about 50,000 INR per month besides the initial investment compared to CuSO_4_ screening method that costs 150-175 INR per month.

## Discussion

For blood collection an appropriate Hb screening method should be available so as to accept as many suitable donors as possible and to prevent any inappropriate deferrals. Any new method to be introduced for Hb screening should save time and expenditure and should be validated against major cell counters or direct cyanmethemoglobin method. It is true the capillary method, unlike venous sampling method of Hb estimation in field conditions for HCS/CuSO4/HemoCue is practicable but as our reference method was based on venous samples, to maintain homogeneity and to have near true values only venous samples were used in this study. Also performance characteristics in terms of sensitivity, specificity and PPV, NPV etc. are better with venous samples as compared to capillary samples. Additionally, as donor acceptance policies are based on venous Hb standards and not on capillary Hb values so venous sampling was preferred over capillary for Hb estimation by all these devices. Not many donors in our study were willing to undergo pre-donation sampling twice. We did not want to give two pricks to our donors (one capillary (finger-prick) and other venous sampling) before actual donation hence we used only single venous sampling in our study population.

Previous studies[8] have used correlation to compare two measurement methods but we decided not to use correlation as it is misleading. Bland-Altman analysis defines, “if two methods are to agree then the mean of the difference between every paired determination will not be statistically different from zero and a limit of agreement can be established”. Our results showed limits of agreement for HCS as 4.7-5.6 g/dl below and above the reference values while HemoCue had values 1.1-1.7 g/dl below and above the reference values which reflected the dispersion of the data around the mean of the difference.

In our study CuSO_4_ method inappropriately passed 6.9% of prospective donors, of which a majority (96.7%) were within 1.0 g/dl of threshold against the reference values, which is quite similar to the observations made by James *et al*.[9] Similarly Boulton *et al*. observed more inappropriate passes by CuSO_4_ method with inappropriate passes being within 1.0 g/dl of the threshold for their gender.[10] However, there are studies with contrasting results as well.[11] This difference in results could be due to use of venous samples in our study. CuSO_4_ has been a traditional way of donor Hb screening despite its limitations. To ensure correct results, CuSO_4_ solution of accurate specific gravity should be used besides taking other technical precautions. Each drop of blood added to the solution affects the specific gravity, therefore changing the solution daily or at least after 25 tests has been recommended.[12]

The CuSO_4_ method has also been found to give inappropriate failures and significant number of such failed donors could be recovered with a revised Hb range or by using an alternative screening method.[13] Using a secondary method of screening, many inappropriate donors could be saved that would otherwise be lost. We found copper sulfate inappropriately deferred 1.0% (10/1014) of the prospective donors in comparison to 0.5% (05/1014) of inappropriate deferral using HemoCue. Considering these results, theoretically HemoCue could have saved 50% of the inappropriately deferred donors, though the number of wrong deferrals by both the methods is low. HemoCue is an easy, rapid and reliable method of donor screening,[14] however its use adds extra expense in a donor screening program if implemented as a primary Hb screening method. It is clear from our results that HemoCue is about 500 times costlier than the CuSO_4_ method and about 40 times costlier than HCS. Although the cost calculations are crude and various other factors viz. cost of lancets, other consumables, electricity charges etc. have not been included, yet implementing HemoCue for primary Hb screening would be beyond reach for many blood centers with limited resources. At the same time, the recovery of inappropriately deferred donors by HemoCue could indicate its usefulness as a secondary screening method.

Hemoglobin color scale did not show good agreement with reference method. HCS gave 25.2% false results against 7.9% by CuSO_4_ and 3.1% by HemoCue, with only 23.2% of the readings within ± 1.0 g/dl. It is true Hb discrimination limits in HCS and CuSO_4_ are different so are not strictly comparable. With the help of Figure 2 (Bland-Altman plots) we have showed limit of agreement for HCS as 4.7-5.6 g/dl below and above the reference values which reflected the data dispersion around the mean of difference. A study by Paddle[15] showed that only 46.08% of readings were correct by HCS and 22.79% results differed more than 2.0 g/dl from the reference value. However, Lewis *et al*.[16] found HCS more reliable than CuSO_4_ for Hb testing in blood donors. The HCS method itself is simple and can easily be carried out by even comparatively inexperienced laboratory workers but it is the most subjective of all the methods and further, it is difficult to compare the intermediate values (e.g. 12.5 g/dl) with HCS. This method may be good to assess the prevalence of anemia in general population in peripheral areas but definitely not suitable for Hb screening in prospective blood donors.

## Conclusion

Out of four different methods for Hb screening we found HCS to be the most subjective method with a large number of inherent errors and thus not appropriate for use in a blood donation setup. HemoCue would be the best method but as mentioned financial constraints would restrict its use as sole screening method. We have provided data that the CuSO_4_ method still stands the test of time and this method can be retained as the primary screening method; however, to save inappropriate deferrals, subsequent testing can be done with HemoCue. This finding could be of value to blood centers with limited resources especially for camp donations where mass donor Hb screening is carried out.
